# Impact of anisotropic conduction and premature atrial contraction on the fractionated atrial potentials

**DOI:** 10.1002/joa3.13161

**Published:** 2024-10-09

**Authors:** Hideko Toyama, Koichiro Kumagai

**Affiliations:** ^1^ Heart Rhythm Center Fukuoka Sanno Hospital Fukuoka Japan; ^2^ International University of Health and Welfare Ōtawara Japan

**Keywords:** atrial fibrillation, fractionated potential, high‐density mapping, premature atrial contraction

## Abstract

**Background:**

Fractionated atrial potential (FAP) during sinus rhythm (SR) may be a new target for ablation of atrial fibrillation (AF). However, the effects of the direction of activation and premature atrial contraction (PAC) on FAP is unknown. Therefore, we examined the impact of anisotropic conduction and PAC on the distribution and areas of FAP.

**Methods:**

FAP map in the left atrium was created in 40 patients with AF before ablation. The distribution and areas of FAP were compared during SR, distal coronary sinus (CS) pacing (S1) and extrastimulus (S2), and paced PAC after SR. FAP was defined as a potential with four or more fragmented deflections.

**Results:**

FAPs during SR were found in the right and mid‐anterior walls and septum in most patients. During S1 compared to SR, FAPs significantly decreased in the right and mid‐anterior walls, appendage, septum, and right inferior wall, while significantly increased in the lateral wall. During S2 compared to S1, FAPs significantly increased in the mid anterior and right and mid posterior walls. During PAC compared to SR, FAPs significantly decreased in the right and mid anterior walls and roof, while significantly increased in the left anterior, left inferior and lateral walls. A rotational activation pattern at the FAP area during CS pacing was observed in 12 patients (30%), mostly in the left inferior wall.

**Conclusions:**

The distribution and areas of FAP vary with anisotropic conduction and extrastimulus. Therefore, FAP should be evaluated not only during SR but also during extrastimulus from the distal CS.

## Graphical abstract

Comparison of FAPs during sinus rhythm, S1 and S2. FAPs were observed in the right anterior wall during sinus rhythm but disappeared during distal CS pacing (S1). On the other hand, FAPs that were not observed in the posterior wall during S1 were newly observed during S2.

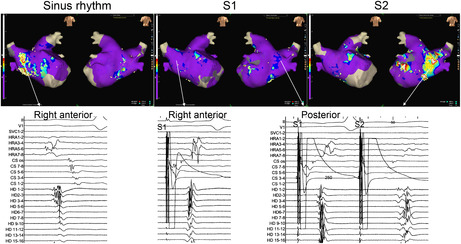

## 1 INTRODUCTION

Pulmonary vein (PV) isolation has been established as an effective treatment for atrial fibrillation (AF).^1^ However, AF can recur from triggers other than the PV. If the origin is reproducibly induced, it can be easily ablated, but focus is not always induced during the procedure, and it is often difficult to identify the true triggers. Hirokami et al. reported that fractionated signal areas in the atrial muscle (FAAM) during sinus rhythm are correlated with the sites of non‐PV foci, ablation of FAAM eliminated non‐PV foci.^2^, ^3^

On the other hand, fractionated atrial potential (FAP) during sinus rhythm represents conduction disorders that cause conduction block and reentry and may be the substrate for AF drivers. Nakatani et al.^4^ demonstrated that ablation of slow conduction areas with delayed potentials or FAPs during sinus rhythm suppressed atrial arrhythmia in patients with nonparoxysmal AF. Thus, FAP during sinus rhythm may be a new target for AF ablation. However, FAP is usually evaluated during sinus rhythm and the effects of the direction of activation and premature atrial contraction (PAC) on FAP are unknown. Therefore, we examined the impact of anisotropic conduction and PAC on the distribution and areas of FAP.

## 2 METHODS

### 2.1 Study population

From October 2023 to April 2024, 45 consecutive patients with all types of AF (28 paroxysmal AF and 17 nonparoxysmal AF) who underwent initial catheter ablation using the EnSite X™ EP System (Abbott) in Fukuoka Sanno Hospital were included. The patients in which sinus rhythm could not be maintained after defibrillation or AF was induced during pacing or mapping were excluded. All antiarrhythmic drugs were discontinued for at least five half‐lives before the study. No patient was taking amiodarone.

### 2.2 Electrophysiological study

A 7F duodecapolar catheter (Bee‐AT, Japan‐Lifeline Co., Ltd., Tokyo, Japan) was inserted into the coronary sinus (CS) from the right internal jugular vein and the distal part was placed around 2 o'clock in the mitral annulus. After the standard transseptal puncture, an Advisor™ HD Grid Mapping Catheter, Sensor Enabled™ (Abbott, St. Paul, MN) and irrigated‐tip ablation catheter (TactiFlex™ CF sensing catheter, Abbott, St. Paul, MN) were inserted into the left atrium (LA) for the mapping and ablation. When AF was sustained, the AF was internally cardioverted.

The programmed atrial stimulation was performed with a basic cycle length of 600 ms from the distal CS, shortening premature atrial stimulation to the atrial refractory period. The distal CS pacing was performed with two different protocols. An extrastimulus was inserted following three consecutive basic stimulations (S1–S2) in 20 patients (protocol‐1), or immediately following sinus rhythm (PAC) in 20 patients (protocol‐2), and at refractory period plus 20 ms coupling intervals.

### 2.3 High‐density mapping

Mapping was performed using the Advisor™ HD Grid Mapping Catheter, Sensor Enabled™ (Abbott, St. Paul, MN) and the EnSite X™ EP System (Abbott) in the whole LA divided into 12 parts including the right, mid, left anterior wall, right, mid, left posterior wall, septum, roof, LA appendage, right and left inferior wall, and lateral wall before ablation. FAP maps are created using the fractionation mapping tool in the EnSite X™ EP on an offline workstation during sinus rhythm, constant pacing, and PAC. Mapping during sinus rhythm and subsequent distal CS pacing was performed simultaneously per point at one time. FAP was defined as a potential with 4 or more fragmented deflections (set at fractionation threshold 4, width 5 ms, refractory 6 ms, and sensitivity 0.04 mV) during sinus rhythm or CS pacing. The distribution and areas of FAP were compared during sinus rhythm, constant distal CS pacing (S1) and extrastimulus (S2), and paced PAC. The voltage map was also assessed to investigate the relationship between FAP and low voltage area (LVA). LVA was defined as an area with bipolar voltage amplitudes of <0.5 mV.

An activation map was also created to assess the rotational activation pattern at the FAP area using omnipolar technology. Omnipolar signals are calculated from a triangular 3‐electrode group called a clique, composed of 3 unipoles, and 2 orthogonal bipoles.^5^ Omnipolar mapping technology has the advantages of both unipolar and bipolar signals, providing a local signal with information on the direction and speed of the wavefront. Electrograms can be calculated at each clique in 360°.^5^ Omnipolar electrograms and assessments of speed and direction are calculated instantaneously, and they are displayed as activation vectors.^5^ The local activation map is overlaid with small arrows omnipolar technology activation vectors map demonstrating the direction of propagation. The rotational activation pattern during sinus rhythm, S1 and S2 and PAC was assessed. The rotational activation pattern was defined as the site showing wavefront propagation rotating >90°at FAP area.

### 2.4 Induction of atrial arrhythmias after ablation

PV isolation or box isolation was performed using an irrigated‐tip ablation catheter (TactiFlex™ CF sensing catheter, Abbott, St. Paul, MN). After ablation, isoproterenol (ISP; 20 μg) was injected intravenously with bolus. Adenosine triphosphate (ATP; 20 mg) was injected intravenously with bolus on a case‐by‐case basis. If spontaneous AF did not occur, rapid pacing for 20 beats while shortening the cycle length by 10 msec from 250 to 180 msec from the proximal CS. When AF was induced, AF was terminated via intracardiac defibrillation, and spontaneous triggers were checked after recovery to sinus rhythm.

### 2.5 Statistical analysis

All statistical data analyses were performed with SAS 9.4 software (SAS Institute Inc., Cary, NC). Continuous variables were compared between groups by the Student's *t*‐test or Wilcoxon rank‐sum test, and data are presented as the mean ± SD. Statistical significance was set at a *p*‐value < .05.

## 3 RESUTLS

### 3.1 Patient characteristics

Of 45 patients, four patients in which sinus rhythm could not be maintained after defibrillation and one patient in which AF was induced during mapping were excluded. Finally, a total of 40 patients (28 paroxysmal AF and 12 nonparoxysmal AF) were included. The baseline patient characteristics are listed in Table 1.

**TABLE 1:** Patient characteristics.

	Protocol‐1 (S1–S2)	Protocol‐2 (PAC)
Number	20	20
Age, years	65.1 ± 9.0	64.8 ± 7.3
Male, *n* (%)	18 (90)	16 (80)
Female, *n* (%)	2 (10)	4 (20)
BMI, kg/m^2^	25.2 ± 3.7	24.7 ± 2.5
CHADS_2_ score	0.9 ± 0.9	1.0 ± 0.8
CHADS_2_‐VASc score	1.4 ± 1.0	1.6 ± 1.2
AF type		
Paroxysmal AF, *n* (%)	14 (70)	14 (70)
Persistent AF, *n* (%)	4 (20)	4 (20)
Longstanding AF, *n* (%)	2 (10)	2 (10)
LA diameter, mm	41.0 ± 5.9	41.5 ± 4.4
LVEF, %	64.7 ± 6.2	64.8 ± 8.8

Abbreviations: AF, atrial fibrillation; LA, left atrium; LVEF, left ventricular ejection fraction.

### 3.2 Comparison of FAP during sinus rhythm, S1 and S2 (protocol‐1)

**FIGURE 1:**
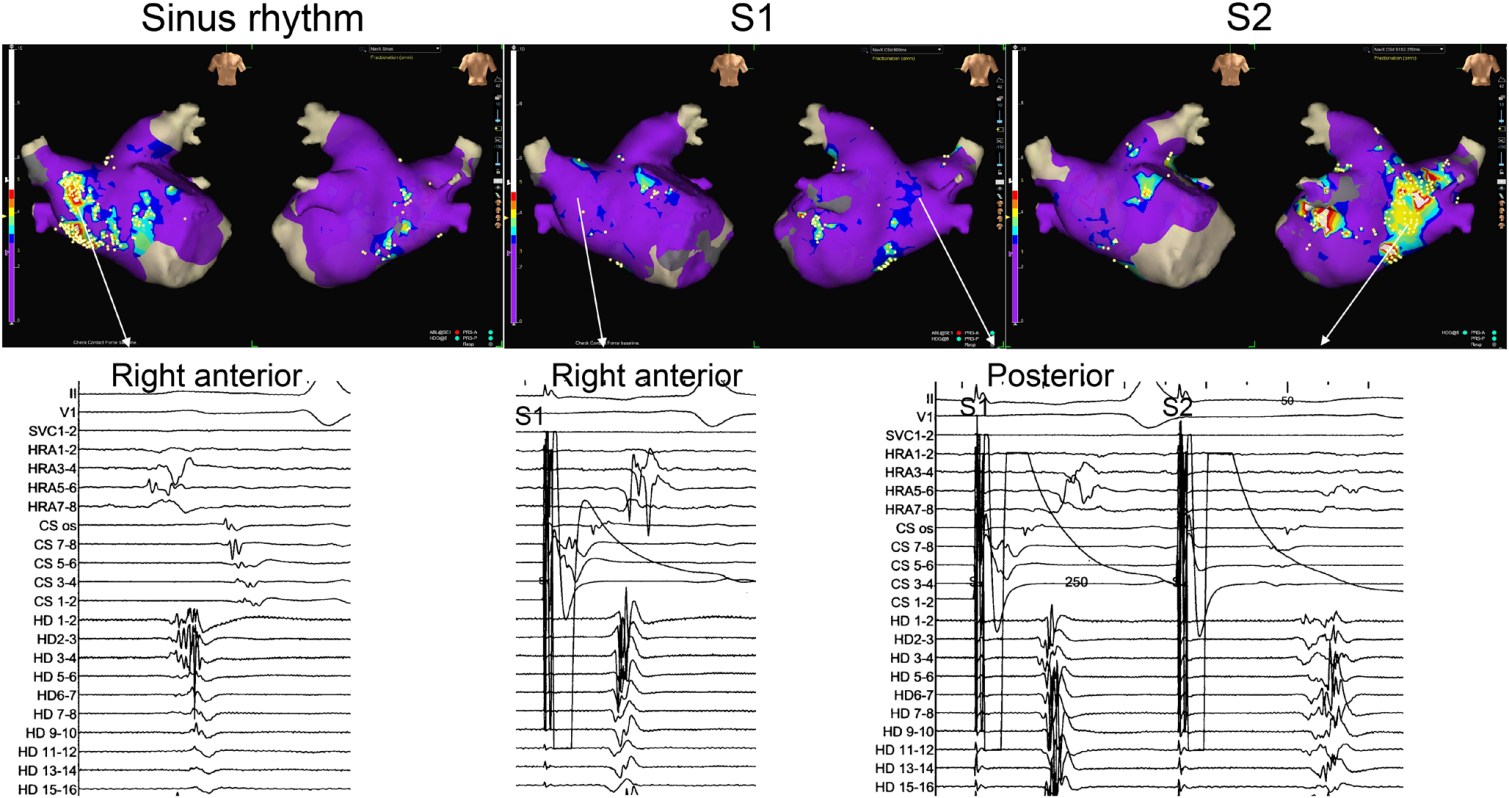
Comparison of FAPs during sinus rhythm, S1 and S2 (protocol‐1). FAPs are shown in white dots. Purple indicates non‐FAP sites. In this case, FAPs were observed in the right anterior wall during sinus rhythm but disappeared during distal CS pacing (S1). On the other hand, FAPs that were not observed in the posterior wall during S1 were newly observed during S2. FAP, fractionated atrial potential.

**FIGURE 2:**
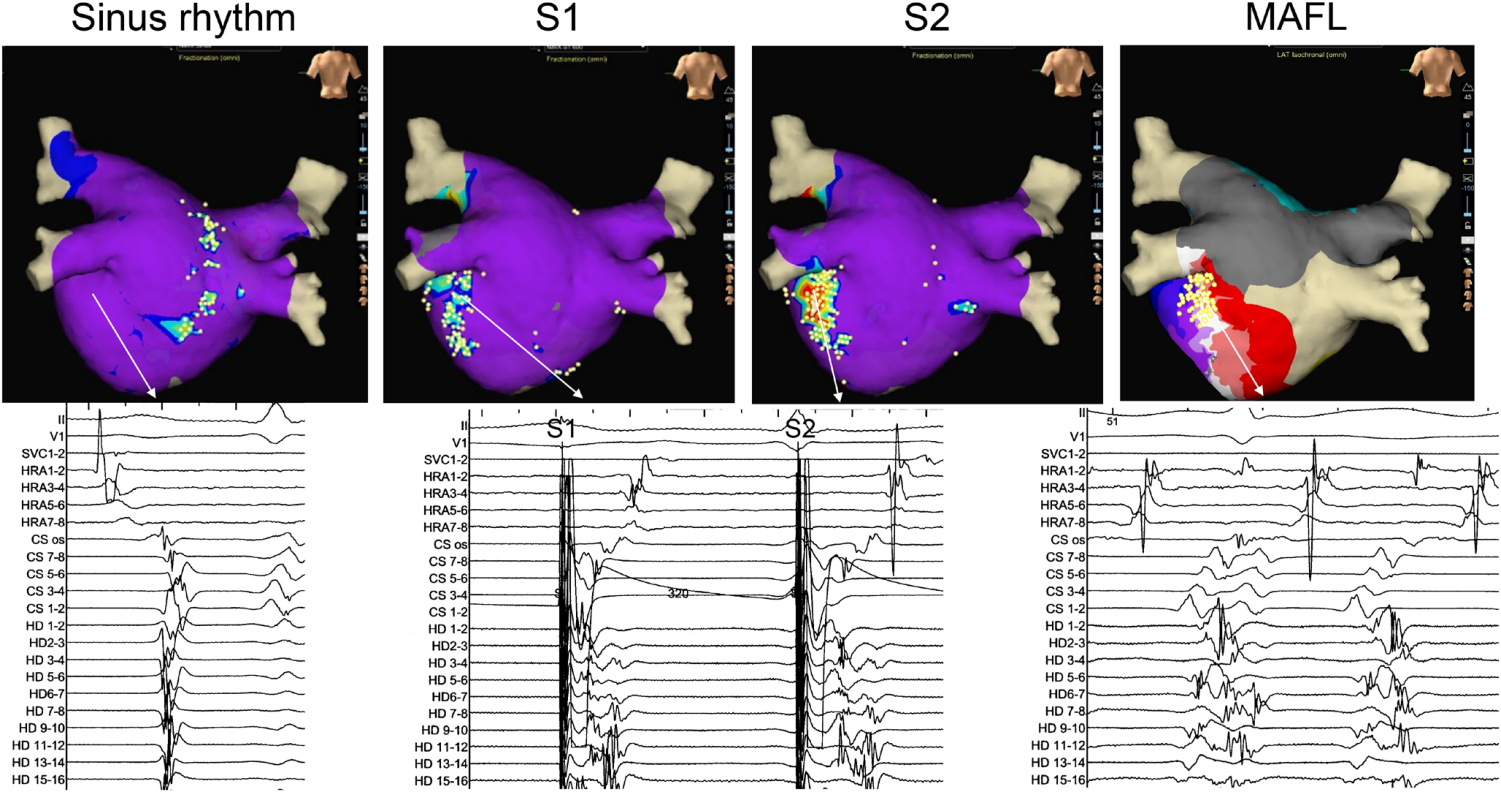
Comparison of FAPs during sinus rhythm, S1 and S2 and mitral flutter. In this case, FAP was not observed in the lateral wall during sinus rhythm but manifested during S1 and S2 pacing. After box isolation, mitral flutter was induced. During mitral flutter, FAPs were observed in the same area. FAP, fractionated atrial potential.

Figure 1 presents a representative example of showing changes in FAP. During sinus rhythm, FAPs were observed in the right anterior wall but disappeared during S1 pacing. On the other hand, FAPs that were not observed in the posterior wall during S1 were newly observed during S2. Figure 2 shows a case in which FAPs were manifested by distal CS pacing. During sinus rhythm, FAP was not observed in the lateral wall, but manifested during S1 and augmented during S2. During mitral flutter induced after box isolation, FAPs similar to that during S2 were observed in the same area.

**FIGURE 3:**
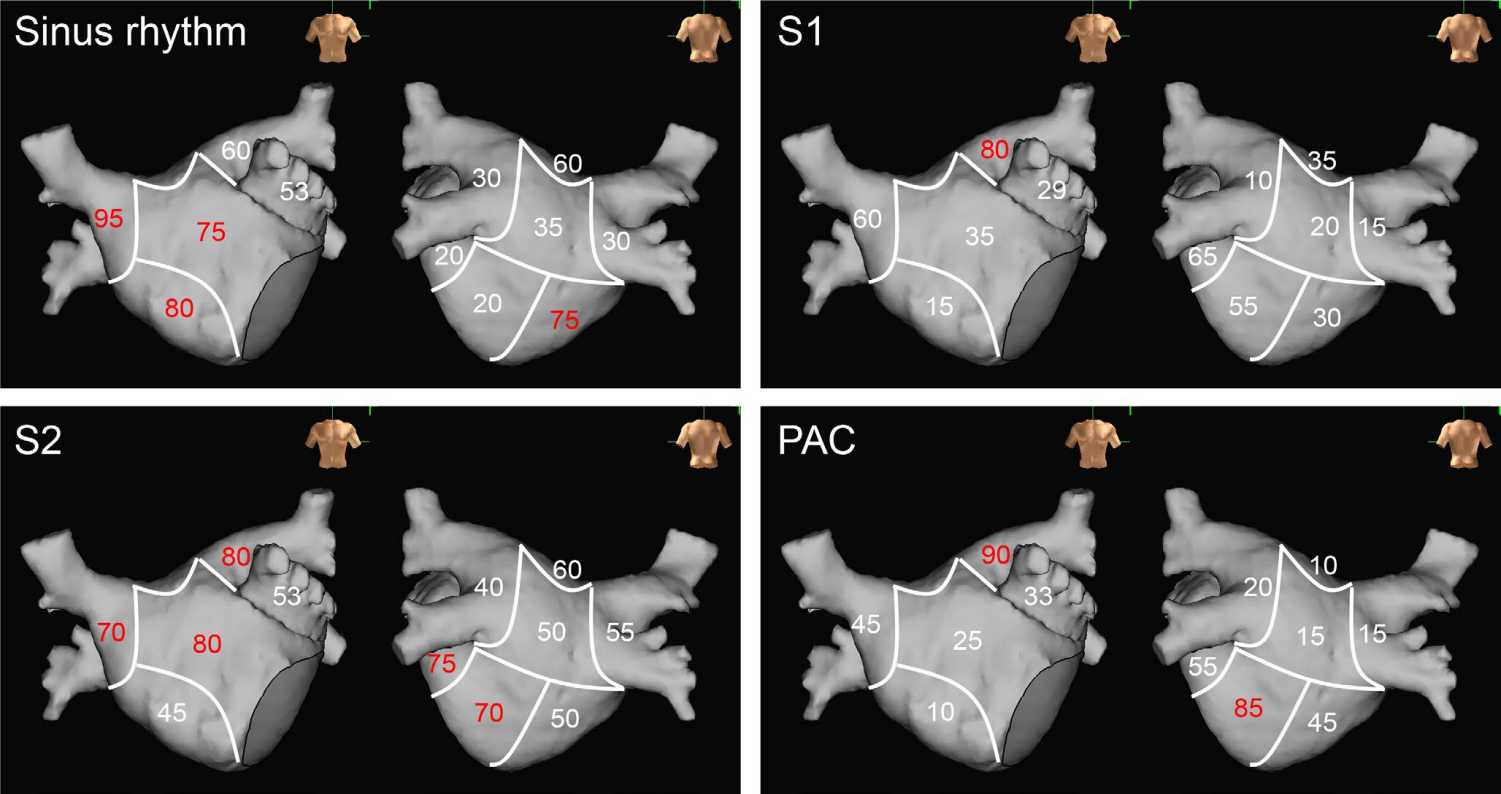
Distribution of FAP for all cases. The number of patients with FAP out of all patients is shown as a percentage. Red indicates greater than 70%. During sinus rhythm, FAPs were mostly found in the right anterior wall (95% of the patients), followed by the septum, mid‐anterior wall, and right inferior wall. During S1, FAPs were found more frequently in the left anterior wall. During S2, FAPs were found more frequently in the mid, left, and right anterior walls, lateral walls, and left inferior walls. During PAC, FAPs were found more frequently in the left anterior wall and left inferior wall. FAP, fractionated atrial potential.

Figure 3 shows the distribution of FAP for all cases. During sinus rhythm, FAPs were mostly found in the right anterior wall (95% of the patients), followed by a septum (80%), mid‐anterior (75%), and right inferior walls (75%). During S1, FAPs were found more frequently in the left anterior wall (80%). During S2, FAPs were found more frequently in the mid (80%), left (80%), and right (70%) anterior, lateral (75%), and left inferior walls (70%).

Table 2 shows the comparison of the FAP area during sinus rhythm, S1 and S2. During sinus rhythm, the FAP area was widest in the right anterior wall. During S1 compared to sinus rhythm, the FAP area significantly decreased in the right and mid‐anterior walls, appendage, septum, and right inferior wall, while significantly increased in the lateral wall. The total area of FAP significantly decreased. During S2 compared to S1, the FAP area significantly increased in the mid anterior wall and right, mid, and left posterior and lateral walls. The total area of FAP significantly increased.

**TABLE 2:** FAP area during sinus rhythm, S1 and S2 (protocol‐1).

	SR	S1	S2	*p* (SR vs. S1)	*p* (S1 vs. S2)
Right anterior, cm^2^	1.5 ± 1.0	0.2 ± 0.3	0.4 ± 0.5	<.0001	.258
Mid anterior, cm^2^	0.6 ± 0.9	0.1 ± 0.3	0.6 ± 0.6	.002	.010
Left anterior, cm^2^	0.7 ± 1.7	0.6 ± 0.7	0.8 ± 1.0	.711	.322
Appendage, cm^2^	0.3 ± 0.5	0.1 ± 0.1	0.2 ± 0.3	.045	.069
Septum, cm^2^	0.4 ± 0.4	0.04 ± 0.1	0.1 ± 0.2	.002	.078
Roof, cm^2^	0.4 ± 0.6	0.1 ± 0.3	0.3 ± 0.5	.115	.056
Right posterior, cm^2^	0.1 ± 0.3	0.1 ± 0.2	0.4 ± 0.7	.438	.020
Mid posterior, cm^2^	0.4 ± 0.8	0.04 ± 0.1	0.3 ± 0.5	.083	.012
Left posterior, cm^2^	0.2 ± 0.8	0.1 ± 0.2	0.2 ± 0.3	.326	.038
Right inferior, cm^2^	0.8 ± 1.1	0.2 ± 0.3	0.3 ± 0.6	.028	.221
Left inferior, cm^2^	0.1 ± 0.2	0.3 ± 0.3	0.4 ± 0.5	.083	.130
Lateral, cm^2^	0.1 ± 0.3	0.4 ± 0.5	0.6 ± 0.7	.003	.043
Total, cm^2^	5.7 ± 4.9	2.2 ± 1.3	4.6 ± 3.1	.039	.0003

Abbreviations: FAP, fractionated atrial potentials; S1, constant pacing from distal coronary sinus; S2, extra stimulation; SR, sinus rhythm.

### 3.3 Comparison of FAP during sinus rhythm and PAC (protocol‐2)

**FIGURE 4:**
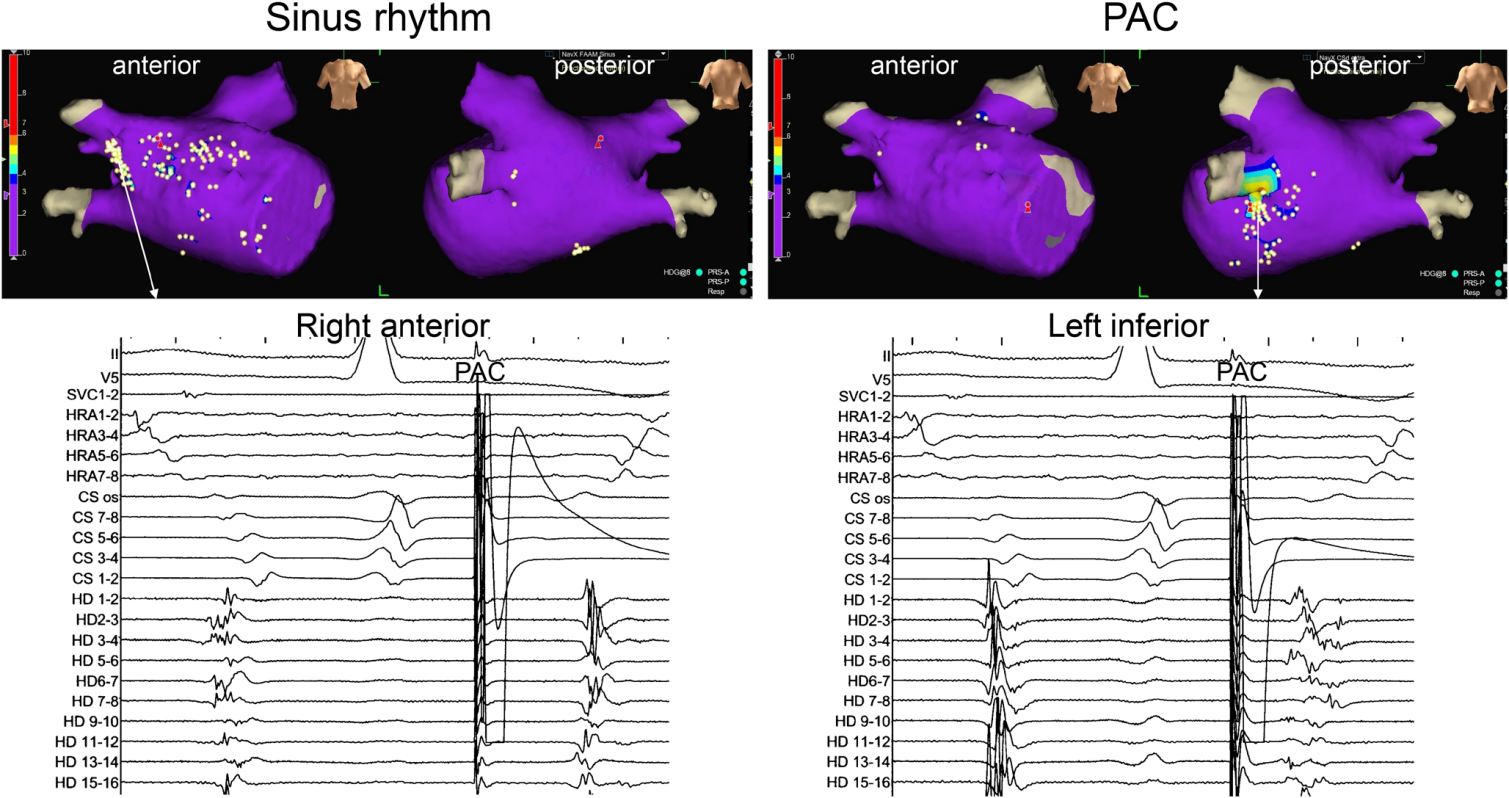
Comparison of FAPs during sinus rhythm and PAC (protocol‐2). In this case, FAPs were observed in the right anterior wall during sinus rhythm but disappeared during PAC. On the other hand, FAPs that were not observed in the left inferior wall during sinus rhythm were newly observed during PAC. FAP, fractionated atrial potential.

Figure 4 presents a representative example of showing changes in FAP. During sinus rhythm, FAPs were observed in the right anterior wall but disappeared during PAC. On the other hand, FAPs that were not observed in the left inferior wall during sinus rhythm were newly observed during PAC.

During PAC, FAPs were found more frequently in the left anterior (90%) and left inferior walls (85%) (Figure 3).

Table 3 shows the comparison of the FAP area during sinus rhythm and PAC. During PAC compared to sinus rhythm, the FAP area significantly decreased in the right and mid anterior walls and roof, while significantly increasing in the left anterior, left inferior, and lateral walls. The total area of FAP significantly decreased.

**TABLE 3:** FAP area during sinus rhythm and PAC (protocol‐2).

	Sinus rhythm	PAS	*p*
Right anterior, cm^2^	1.8 ± 1.5	0.2 ± 0.3	<.0001
Mid anterior, cm^2^	0.6 ± 0.4	0.2 ± 0.3	<.0001
Left anterior, cm^2^	0.2 ± 0.3	0.5 ± 0.4	.001
Appendage, cm^2^	0.2 ± 0.5	0.2 ± 0.3	.601
Septum, cm^2^	0.2 ± 0.3	0.1 ± 0.2	.104
Roof, cm^2^	0.3 ± 0.3	0.1 ± 0.2	.003
Right posterior, cm^2^	0.1 ± 0.2	0.04 ± 0.1	.127
Mid posterior, cm^2^	0.1 ± 0.1	0.1 ± 0.1	.797
Left posterior, cm^2^	0.1 ± 0.2	0.1 ± 0.1	.471
Right inferior, cm^2^	0.3 ± 0.4	0.2 ± 0.4	.275
Left inferior, cm^2^	0.1 ± 0.2	0.5 ± 0.5	.001
Lateral, cm^2^	0.02 ± 0.1	0.5 ± 0.7	.002
Total, cm^2^	3.9 ± 2.2	2.5 ± 1.5	.002

Abbreviations: FAP, fractionated atrial potentials; PAC, premature atrial contraction.

### 3.4 Relationship between FAP and LVA

The FAP and LVA sites were coincident in five patients (25%) during sinus rhythm, six patients (30%) during S1, 10 patients (50%) during S2, and four patients (20%) during PAC. FAPs in the LVA had more fragmented deflections than those in the normal voltage area in 6 patients, fewer in 8 patients, and no difference in 6 patients.

### 3.5 Rotational activation pattern in FAP area

**FIGURE 5:**
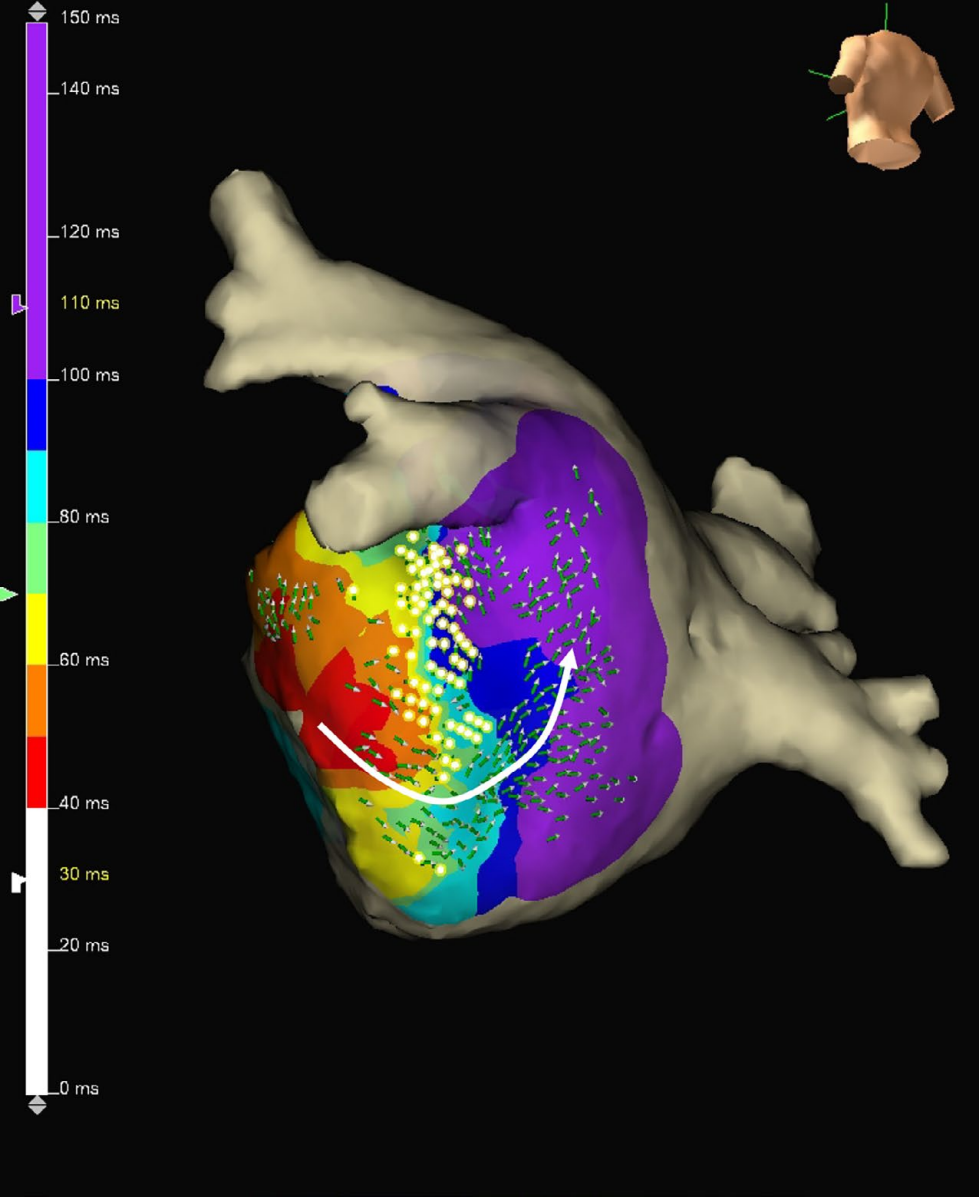
Rotational activation pattern in FAP area. In this case, a rotational activation pattern was observed around the FAP area in the left inferior wall during S2. FAP, fractionated atrial potential.

Figure 5 presents a representative example of showing a rotational activation pattern around the FAP area in the left inferior wall during S2. Such a pattern was not observed during sinus rhythm but observed in three patients during S1, five patients during S2, and four patients during PAC (total 12%; 30%). The rotational activation pattern was observed in the left inferior wall in nine patients, the left anterior wall in one, the right posterior wall in one, and the mid anterior wall in one.

Mitral flutter was induced in two cases in whom the rotational activation pattern was found in the left inferior wall.

### 3.6 Induction of atrial arrhythmias after ablation

PV isolation alone was performed in nine patients with paroxysmal AF and box isolation was performed in 19 patients with paroxysmal AF and 12 patients with nonparoxysmal AF. After ablation, a bolus injection of ISP did not induce non‐PV triggers in all patients. ATP was used in 12 patients but did not induce non‐PV triggers. Rapid pacing‐induced AF in two patients, but spontaneous triggers did not occur after defibrillation. Rapid pacing‐induced atrial flutter in eight patients (typical flutter in six and mitral flutter in two) and focal atrial tachycardia from the superior vena cava (SVC) in one patient. After cavo‐tricuspid isthmus ablation, mitral isthmus ablation, and SVC isolation, atrial arrhythmias were no longer induced.

## 4 DISCUSSION

### 4.1 Main findings

The present study demonstrated that (1) FAPs during sinus rhythm were found in the right anterior walls in most patients, (2) during S1 compared to sinus rhythm, FAPs significantly decreased in the right and mid‐anterior walls, appendage, septum, and right inferior wall, while significantly increased in the lateral wall. During S2 compared to S1, FAPs significantly increased in the mid anterior and right and mid posterior walls, (3) during PAC compared to sinus rhythm, FAPs significantly decreased in the right and mid anterior walls and roof, while significantly increased in the left anterior, left inferior, and lateral walls, and (4) the rotational activation pattern that was not observed during sinus rhythm was found in 30% of the patients during the distal CS pacing, mostly in the left inferior wall.

### 4.2 Effect of direction of activation on FAP

Remodeling of intercellular connections and tissue damage result in a discontinuous distribution of conduction properties, which is heterogenous anisotropy.^6^, ^7^ In such sites, conduction disorders are direction‐dependent and electrograms recorded in these areas may be fractionated. FAP can be formed by the various directions, fiber orientation, and complex arrangement of the myocardium. FAP may represent the heterogeneity of anisotropic tissue.^6^ The changes in anisotropic properties promote slow conduction and unidirectional block. We showed that FAP observed during sinus rhythm disappeared during distal CS pacing, and conversely, FAP not observed during sinus rhythm newly appeared during distal CS pacing. Thus, a significant site of conduction disorders would be missed when performing FAP mapping during sinus rhythm alone, therefore, FAP mapping during distal CS pacing could also be considered.

### 4.3 Effect of PAC on FAP

PAC triggering AF most often originates from within the PVs.^8^ Therefore, we performed premature atrial stimulation from the distal CS close to the left side PVs. PAC was inserted with pacing immediately after sinus rhythm to approximate clinical PAC in protocol‐2. Previous studies have shown that the widening of the fragmented atrial activity zone by premature atrial stimulation is related to AF vulnerability.^9^, ^10^ Spach et al.^11^ showed that anisotropy is direction‐dependent, as premature stimulation in heterogeneous tissue resulted in unidirectional longitudinal conduction blocks or zigzag longitudinal conduction. In the present study, FAPs that were hidden during sinus rhythm were manifested during extrastimulus from the distal CS. Schie et al.^6^ also demonstrated that PAC from the opposite direction during sinus rhythm increases fractionation. PACs provoke conduction disorders in nonuniform anisotropic tissue, thereby initiating reentry.^6^, ^7^ As shown in Figure 5, the rotational activation pattern was observed at the FAP area during extrastimulus. Premature atrial stimulation can be done with either S1–S2 or PAC. The total area of FAP was increased more in S1–S2 than in PAC compared to sinus rhythm. FAP and LVA sites were more coincident during S2 compared to PAC. Thus, S1–S2 may be better than PAC for evaluating FAP.

### 4.4 Ablation targeting FAP

Hirokami et al.^12^ demonstrated that ablation of FAAM, which is correlated with the sites of non‐PV foci, significantly decreased the AF recurrence rate compared with non‐FAAM ablation in patients with recurrent AF after multiple sessions. We did not perform additional FAP ablation, because the non‐PV foci were not induced from the FAP area after ablation. This may be because our induction method was not as aggressive as theirs, many of the cases were paroxysmal AF, and box isolation was performed in many cases. FAPs were found in the right anterior wall in almost all patients, but not all FAPs necessarily trigger AF. Thus, the potential at the non‐PV origin site may be FAP, but the reverse is not true.

Nakatani et al.^4^ demonstrated that Ripple map‐guided ablation of slow conduction areas with delayed potentials or FAPs during sinus rhythm significantly increased the free rate from atrial arrhythmia compared to the empirical linear ablation. We found a rotational activation pattern at the FAP area during distal CS pacing, but we did not perform ablation of such sites except in two cases with induced mitral flutter. When a rotational activation pattern was present in the anterior PV wall, inferior wall, or posterior wall, it may have been affected by box isolation. We have reported that the posterior wall box isolation decreases the rotors and multiple wavelets in the anterior and inferior walls.^13^

There are preferred sites of FAP that are seen in most cases during sinus rhythm. Should all these FAPs be targeted for ablation? Since FAPs during extrastimulus are more related to LVA and rotational activation patterns, those FAPs might be a better target for ablation. However, it is unclear whether ablation targeting FAPs manifested by extrastimulus would improve outcomes.

### 4.5 Limitations

First, we did not perform additional FAP ablation, because AF was not initiated from the FAP area after ablation. Therefore, whether ablation targeting FAPs manifested by extrastimulus improves outcomes needs to be examined in future studies. Second, we performed the pacing from distal CS alone, the change of FAP by other pacing sites (e.g., proximal CS pacing) is unclear. Third, we did not perform FAP mapping in the right atrium, the impact of anisotropic conduction and PAC on the right atrial FAP is unclear. Finally, different 3D mapping systems have different definitions of FAP, which would affect the interpretation of FAP.

## 5 CONCLUSION

The distribution and area of FAP varies with anisotropic conduction and PAC. Therefore, FAP should be evaluated not only during sinus rhythm but also during extrastimulus from the distal CS.

## CONFLICT OF INTEREST STATEMENT

The authors have no conflicts of interest associated with this manuscript.

## ETHICS STATEMENT

Our study was approved by the Fukuoka Sanno Hospital's Institutional Review Board.

## PATIENT CONSENT STATEMENT

Informed consent was obtained in the form of opt‐out on the website.
